# Body Surface Gastric Mapping Delineates Specific Patient Phenotypes in Adolescents With Functional Dyspepsia and Gastroparesis

**DOI:** 10.1111/nmo.70018

**Published:** 2025-03-19

**Authors:** Gayl Humphrey, Celia Keane, Gabriel Schamberg, Alain Benitez, Stefan Calder, Xu Binghong, Christian Sadaka, Christopher N. Andrews, Greg O’Grady, Armen Gharibans, Hayat Mousa

**Affiliations:** ^1^ Department of Surgery, Faculty of Medical and Health Sciences The University of Auckland Auckland New Zealand; ^2^ Alimetry Ltd. Auckland New Zealand; ^3^ Division of Gastroenterology The Children's Hospital of Philadelphia Philadelphia Pennsylvania USA; ^4^ Perelman School of Medicine University of Pennsylvania Philadelphia Pennsylvania USA; ^5^ The Division of Gastroenterology, Cumming School of Medicine University of Calgary Calgary Alberta Canada

**Keywords:** disorders of gut–brain interaction (DGBI), gastric alimetry, gastroduodenal symptoms

## Abstract

**Background:**

Diagnosing pediatric patients with chronic gastroduodenal symptoms is clinically challenging, with the role of gastric emptying testing being controversial. Body Surface Gastric Mapping (BSGM) is a new diagnostic test that can identify specific patient phenotypes in adults with gastric dysfunction. This study evaluates whether BSGM can delineate specific phenotypes in adolescents and provide clinically meaningful distinctions between gastroparesis and functional dyspepsia diagnoses.

**Methods:**

A prospective cross‐sectional study recruited adolescents aged 12 to 21 between 2022 and 2024. Controls were recruited from New Zealand and patients from the Children's Hospital of Philadelphia, USA. BSGM followed a standardized protocol, including simultaneous symptom reporting and completion of validated symptom, psychometric, and physical health questionnaires.

**Key Results:**

Fifty‐six subjects were recruited (31 controls, 25 patients); median age 16; 96% of patients were female. Control data showed that adult reference intervals provided an acceptable interpretation framework. Patients with FD (*n* = 10) and gastroparesis (*n* = 15) had common symptoms, mental health, quality of life, and functional disability (all *p* > 0.05). Three distinct BSGM phenotypes were identified: *BSGM Normal* (*n* = 10), *BSGM Delay* (*n* = 8), and *Low Stability/Low Amplitude* (*n* = 7), having spectral differences in BMI‐Adjusted Amplitude 34.6 versus 39.1 versus 19.9 (*p* = 0.01) and Gastric Alimetry Rhythm Index: 0.45 versus 0.45 versus 0.19 (*p* = 0.003). BSGM phenotypes demonstrated differences in symptoms (nausea *p* = 0.04), physical health (*p* = 0.04), and psychometrics (anxiety *p* = 0.03).

**Conclusion and Inferences:**

Adolescents with FD and gastroparesis have overlapping clinical profiles, making treatment challenging. Conversely, employing BSGM to categorize patients into distinct phenotypes reveals clinically relevant differences, offering avenues for individualized therapeutic pathways.

## Graphical abstract

Persistent symptoms such as nausea and abdominal pain are prevalent in the pediatric population and signficantly impact their well‐being. The overlapping nature of these symptoms makes distinguishing differences clinically difficult. Using Body Surface Gastric Mapping, specific phenotypes were identified thereby improving promise to enhance clinical decision‐making.

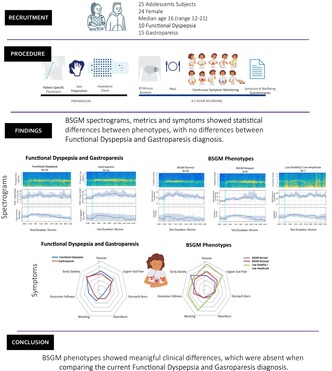

**Summary**

- Gastric emptying testing (GET) is used to differentiate FD and gastroparesis patients, yet these disorders show overlapping clinical characteristics. BSGM combines a non‐invasive gastric electrophysiological mapping test with validated symptom profiling to improve patient subgroup phenotyping.
- Adolescent FD and gastroparesis patients defined by GET and Rome IV were indistinguishable by symptoms, quality of life, and health psychology. In contrast, BSGM differentiated FD and gastroparesis patients into three distinct phenotypes with meaningful clinical differences.
- BSGM improves patient differentiation by identifying discrete subgroups of patients with specific dysmotility profiles, with superior symptom and biopsychosocial correlations. These subgroups have implications for diagnoses and tailoring of treatment and management decisions.

**Abbreviations**
BSGM: body surface gastric mapping
FD: functional dyspepsia
ff‐AR: fed‐to‐fasted amplitude rati
GA‐RI: gastric alimetry rhythm index
GET: gastric emptying test

## 1 Introduction

Persistent upper gastroduodenal symptoms such as nausea, vomiting, bloating, and abdominal pain are prevalent in the pediatric population [1], impacting quality of life and leading to frequent healthcare presentations [2, 3]. The Rome IV pediatric criteria provide a diagnostic framework to support a positive diagnostic approach [4]; however, overlapping symptoms and diagnostic criteria continue to pose challenges to personalized treatment [5].

Per Rome IV, FD is subclassified into postprandial distress syndrome (PDS) and epigastric pain syndrome (EPS) [4], yet approximately 35% of FD patients experience both PDS and EPS symptoms [6]. Confirmed delayed gastric emptying is used to differentiate between patients with FD or gastroparesis, as they often present with similar symptoms [7]. While a delayed gastric emptying test is the gold standard for confirming gastroparesis, studies have shown that up to 25% of patients with FD also exhibit delayed gastric emptying [8], thus blurring the distinction between these two groups and underscoring an overlapping pathophysiology. These overlaps between FD and gastroparesis underscore the limitations of gastric emptying studies, suggesting they may not be as diagnostically definitive as once believed [9, 10, 11]. New gastric tests providing actionable biomarkers are needed to better discriminate disease pathophysiology to inform care.

Body Surface Gastric Mapping (BSGM) (Gastric Alimetry, Alimetry, New Zealand) is a new diagnostic test involving high‐resolution electrophysiology coupled with simultaneous symptom profiling. Essential developments, such as a large single array with a dense grid of embedded electrodes to accommodate weak signals and individual anatomical differences, plus a validated sophisticated artifact detection algorithm capable of filtering noise from gastric signals [12], are implemented in BSGM to make it a substantially superior test compared to legacy electrogastrography [13, 14, 15]. The array also has the adaptability to have one to two electrode rows removed to better fit smaller torsos without compromising signal acquisition or quality. The latter feature reduces overlapping frequency ranges of activity produced by the stomach and colon. Although, despite overlapping gastric and colonic operating frequencies, the colonic signal morphology over longer periods differs significantly from that of the stomach [16]. Specifically, the stomach produces sustained contractions at a stable frequency over timescales (often hours) much longer than contractions produced by the colon, which are not typically concentrated around a single frequency [17].

Several studies applying BSGM in adults have demonstrated the capability to phenotype specific disease subgroups in gastroduodenal disorders [18, 19]. A recent study also demonstrated that using BSGM phenotypes in clinical practice aided decision‐making and reduced healthcare costs [20]. In addition, in a large head‐to‐head study, BSGM demonstrated a higher yield for gastric motility abnormalities than gastric emptying testing (GET) alone, with improved correlations to symptoms and psychometrics [9].

Feasibility studies using BSGM in pediatrics have been presented in the abstract form, suggesting that BSGM is acceptable, feasible, and safe for children as young as five [21, 22].

This study is the first detailed report on using BSGM with a pediatric population, focusing on adolescents. The aims were to define whether BSGM can delineate specific patient subgroups within FD and gastroparesis and to compare symptoms, health psychology, and quality of life across these subgroups.

## 2 Methods

This was a pragmatic cross‐sectional study, with a sample size estimated at a minimum of 50 subjects being sufficient to evaluate group‐level differences [23], which is supported by recent feasibility studies in pediatric populations [21, 22]. The study is reported in accordance with the STROBE guidelines [24].

### 2.1 Population

Adolescent participants were recruited from Auckland, New Zealand (controls), and the Children's Hospital of Philadelphia, USA (patients). Ethics was approved by the relevant institutional review boards, and all participants provided informed consent and parent assent. Healthy controls were recruited through local advertising and patient participants through clinical referrals. Eligibility criteria were that participants were aged between 12 and 21 years, had a body mass index (BMI) < 35 kg/m [2] (as per the BSGM information for use recommendations), and had no history of gastric surgery other than percutaneous enteric gastrostomy tube insertion (allowed for patients). Patient eligibility also included a diagnosis of FD (confirmed by Rome IV and a normal GET) or a clinical diagnosis of gastroparesis (confirmed by a delayed GET). All GET were undertaken within 24 months of the BSGM test. All participants completed the 4.5‐h BSGM test.

### 2.2 Procedures

Participant demographics, anthropometric measures, and clinical data, including GET outcomes, were obtained. A delayed GET outcome confirmed gastroparesis, and Rome IV criteria and a normal GET confirmed FD [25, 26]. All participants completed age‐appropriate validated questionnaires for gastroduodenal symptoms [27], functional disability [28], mental well‐being, [29, 30] and quality of life [31] on the day of the BSGM test.

**FIGURE 1:**
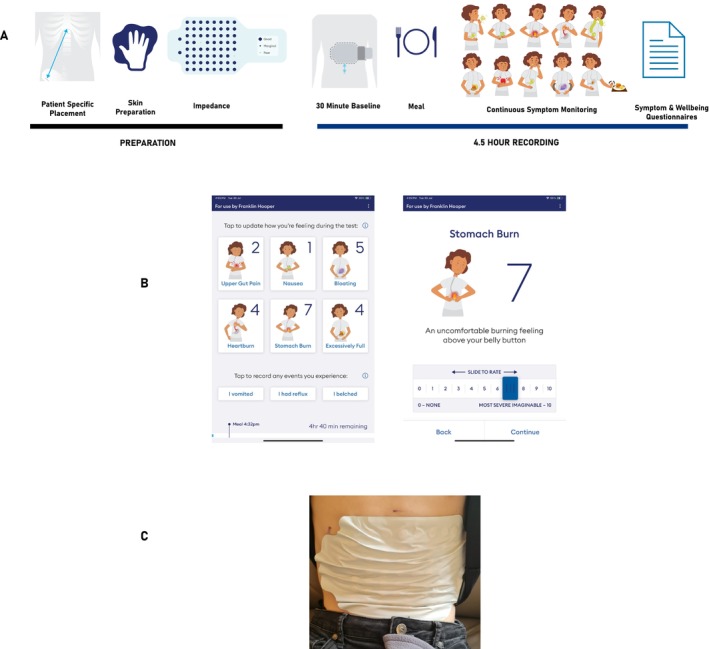
(A) The BSGM protocol encompassing a 30‐min fasting baseline, a 10‐min meal challenge, and a 4‐h postprandial recording with concurrent symptom logging at 15‐min intervals and completion of validated questionnaires. (B) Screenshots of the symptom‐logging app. (C) Image of an array and reader on the abdomen of an adolescent.

Figure 1A presents the standardized BSGM protocol using the Gastric Alimetry system (details previously published [32]), screenshots of the symptom‐logging App (Figure 1B), and an example of the BSGM array on an adolescent's abdomen (Figure 1C). The standard test protocol involved gentle skin exfoliation with an electrolyte gel, a 30‐min fasting baseline, a standardized meal (68 g Clif bar; 250 kCal with 45 g carbohydrate, 9 g protein, 5 g fat, 4 g fiber), and 200 mL of water, consumed over 10 min, followed by a 4‐h postprandial recording. Simultaneously, all participants reported their symptoms on the Gastric Alimetry App using an 11‐point Likert scale of 0 = ‘none’ to 10 = ‘worst symptom imaginable’ and reported episodic symptoms (belching, reflux, and vomiting) at a minimum of 15‐min intervals. The total symptom burden score and individual symptom scores were calculated and averaged [33].

BSGM spectral metrics are reported as Principal Gastric Frequency (PGF), BMI‐Adjusted Amplitude, Gastric Alimetry Rhythm Index (GA‐RI) and fed‐fasted Amplitude Ratio (ff‐AR) (for more detailed and technical information, refer to [12, 32, 34]).

In the absence of validated pediatric normative reference intervals, abnormalities were defined using adult reference intervals [18, 32]. Visual assessment and interpretation of each participant's spectrogram and spectral plot, as well as a detailed review of the signal traces for assessment of extrinsic (movement) and intrinsic (e.g., colonic signal) artifact, was also undertaken to support accurate report interpretation. Additionally, the adolescent control data generated in this study were compared to adult normative ranges to ensure that these provided an acceptable provisional interpretative framework. Patients were then phenotyped a priori according to abnormalities identified in the BSGM metrics, their symptom profile, and meal response pattern based on previous approaches used in adults [16, 19].

### 2.3 Analysis

Statistical analyses were performed using SPSS version 29.0 (IBM Corporation, Armonk, NY). Variables were summarized with descriptive statistics, normality was assessed, and data were expressed as median and interquartile range (IQR). Statistical comparisons were performed between study groups using Student's *t*‐test, Pearson's chi‐squared tests, Mann–Whitney *U*, Kruskal‐Wallis, or Fisher's exact tests as appropriate. Pearson's correlation coefficient and Spearman's correlation were also applied, as appropriate, to the data type.

## 3 Results

Fifty‐six subjects comprising 31 healthy controls and 25 patients were recruited; median age 16 years (range 12–20); 80% were female and 74% Caucasian (Table 1). Median BMI was not different between the adolescent groups (*p* = 0.2). Summary patient and control data for total symptom burden score, quality of life, and mental well‐being are presented in Table 2. They are similar to those reported in other international studies [35].

**TABLE 1:** Pediatric healthy control and patient demographics.

Variables	Healthy controls (*N* = 31)	Patients (*n* = 25)	*p*
Demographics			
Sex (M [%])	19 (61)	1 (4)	< 0.001
Age years (median [IQR])	14.7 (13–16)	16.1 (14–18)	0.02
Ethnicity (*N* (%)			
White/European	22 (71)	20 (80)	—
Māori^a^	1 (3)	—	—
Pacific/Hawaiian	2 (6.5)	—	—
Black/African American	2 (6.5)	2 (8)	—
Hispanic/Latinx	—	3 (12)	—
Chinese	2 (6.5)	—	—
Other	2 (6.5)	—	—
BMI Kg/m^2^ (median [IQR])	19.7 (17.4–21.2)	20.7 (19.0–22.7)	0.2
Diagnosis			
Gastroparesis (*n*)	—	15	—
Functional dyspepsia (*n*)	—	10	—
Quality (median [IQR])			
% of Meal completion	100	66.7 (40–100)	< 0.001
% Artifact	26.3 (16.3–42.0)	21.9–12.85‐29.1)	0.6
Impedance	108.9 (67.4–124.2)	131.2 (91.3–178.7)	0.04

^a^ Māori: Indigenous population of New Zealand.

**TABLE 2:** Total symptom burden and individual symptom scores, clinical symptoms, quality of life, functional disability, and mental well‐being questionnaire outcomes for healthy controls and patients.

Variables (median [IQR])		Healthy controls	Patients	*p*
		*N* = 31	*N* = 25	
Total symptom burden score		1.2 (0.0–3.6)	16.4 (5.3–33.1)	< 0.001
Individual symptoms				
Early satiety		1 (0.0–2.0)	4 (1.0–6.0)	0.04
Excessively full		0 (0.0–0.55)	2.55 (0.18–5.7)	< 0.001
Bloating		0	1.8 (0.0–3.95)	< 0.001
Heartburn		0	0.3 (0.0–2.45)	< 0.001
Nausea		0	2.3 (0.15–6.5)	< 0.001
Upper gut pain		0	2.8 (0.03–5.2)	< 0.001
Stomach burn		0	1.2 (0.0–4.3)	< 0.001
Patient completed questionnaires				
Gastrointestinal symptoms	↓	89.8 (83.4–95.6)	53.72 (38.51–70.61)	0.02
Nausea severity scale	↑	0.0 (0.0–14.0)	20.4 (10.2–29.7)	0.003
Abdominal pain index	↑	0	23.0 (14.2–31.0)	< 0.001
Functional disability index	↑	0.0 (0.0–1.0)	20.0 (9.0–30.25)	< 0.001
Quality of life	↓	97.0 (85.0–100)	57.0 (42.0–71.0)	< 0.001
Anxiety	↑	38.0 (33.5–52.5)	53.6 (43.2–59.5)	< 0.001
Depression	↑	43.8 (33.5–52.5)	50.9 (43.5–59.5)	0.32

*Note:* ↑↓ indicates the direction of severity outcome for each questionnaire.

The previously developed adult reference intervals [32, 34] were compared to 31 healthy control adolescents aged 12–18 years to verify the acceptability of their use in adolescents. The main difference was that pediatric controls showed a modestly lower median GA‐RI than adults (0.35 [0.22–0.43] vs. 0.50 [0.39–0.64], *p* < 0.001; Table 3). Analyzing GA‐RI over time shows that the pediatric healthy control cohort has a short and sharp meal response with a quicker return to baseline or fasting state than their adult counterparts (refer to Figure S1, which presents the Pediatric and Adult average spectrograms and Figure S2, which presents the pediatric and adult median GA‐RI metrics over time as a line graph).

**TABLE 3:** Comparison between pediatric and adult controls for average BMI‐adjusted amplitude, principal gastric frequency, gastric alimetry rhythm index, fed‐toto‐fasted amplitude ratio, and meal response ratio.

BSGM spectral metrics (median [IQR])	Pediatric controls	Adult controls	*p*
	*N* = 31	*N* = 110	
BMI‐adjusted amplitude	33.90 (28.9–40.9)	36.90 (29.6–50.7)	0.11
PGF	3.00 (2.86–3.19)	3.05 (2.90–3.21)	0.58
GA‐RI	0.35 (0.22–0.43)	0.50 (0.39–0.64)	< 0.001
ff‐AR	2.12 (1.79–2.77)	1.84 (1.25–2.21)	0.02
Meal response ratio	1.44 (1.32–1.82)	1.10 (0.94–1.38)	< 0.001

Abbreviations: ff‐AR, fed‐to‐fasted amplitude ratio; GA‐RI, Gastric Alimetry hythm index; PGF, principal gastric frequency.

The objective of this study was to identify group‐level distinctions, with the observed overall similarity between the spectral metrics of pediatric and adult control groups providing confidence that the application of adult reference intervals a priori could effectively differentiate phenotypes and yield significant group‐level differences within the pediatric patient cohort.

### 3.1 Gastroparesis Versus Functional Dyspepsia

**FIGURE 2:**
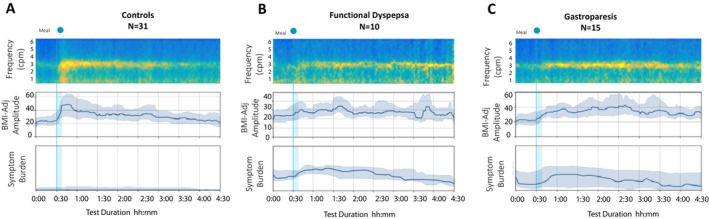
Averaged BSGM spectral and symptom burden plots with median (IQR shaded) for BMI‐adjusted amplitude and symptom burden for Contols (A), Functional Dyspepsia (B) and Gastroparesis (C).

All 25 patients underwent GET, with 15 having gastroparesis and 10 having normal GET and positive for FD according to Rome IV criteria. There were no significant differences in total symptom burden, individual symptoms reported during the test, or clinical symptoms, quality of life, functional disability, or mental well‐being questionnaire outcomes between patients with gastroparesis or FD. Likewise, the BSGM metrics were not significantly different between gastroparesis or FD patient groups (Table 4). Figure 2 presents the averaged spectral and symptom burden plots derived from the Gastric Alimetry BSGM test for healthy controls and patients with FD and gastroparesis.

**TABLE 4:** BSGM within‐test total symptom burden and individual symptom scores; clinical symptoms, quality of life, functional disability, and mental well‐being questionnaire outcomes, and BSGM spectral metric outcomes for functional dyspepsia and gastroparesis patients.

Variables (median [IQR])		Functional dyspepsia	Gastroparesis	*p*
		*n* = 10	*n* = 15	
Within‐test reported symptoms				
Total symptom burden score		27.50 (15.7–33.2)	22.8 (5.0–39.4)	0.95
Individual symptoms				
Early satiety		5 (1.0–7.0)	4.1 (3–6)	0.48
Excessively full		2.7 (2.4–6.5)	1.3 (0.1–5.8)	0.8
Bloating		3.5 (1.8–4)	1.8 (0.5–4.9)	0.55
Heartburn		0.5 (0.0–2.0)	0.5 (0.0–4.1)	0.34
Nausea		3.6 (2.3–5.9)	4.5 (0.3–7.1)	0.79
Upper Gut Pain		4.3 (2.0–5.2)	2.2 (0.0–6.7)	0.88
Stomach Burn		0.3 (0.0–5.0)	1.1 (0.0–4.6)	0.39
Patient‐completed questionnaires				
Gastrointestinal symptoms module	↓	54.2 (33.1–68.5)	47.3 (38.5–71.2)	0.57
Nausea severity scale	↑	27.0 (19.7–29.2)	30.0 (20.0–32.0)	0.71
Abdominal pain index	↑	25.0 (12.5–33.0)	25.1 (15.0–30.0)	0.34
Functional disability index	↑	23.5 (11.7–35.0)	27.0 (9.0–34.0)	0.88
Quality of life	↓	60.0 (45.0–74.5)	53.0 (47.0–63.0)	0.16
Anxiety	↑	58.3 (43.3–61.8)	54.8 (47.2–61.8)	0.58
Depression	↑	50.7 (43.5–65.1)	49.8 (37.7–62.6)	0.48
BSGM spectral data				
Principal Gastric Frequency (cpm)		2.90 (2.84–2.99)	3.05 (2.95–3.13)	0.21
BMI‐adj Amplitude (μV)		26.6 (24.5–31.8)	40.4 (27.9–48.2)	0.06
Gastric Alimetry Rhythm Index		0.31 (0.19–0.36)	0.47 (0.32–63)	0.13
Fed‐to‐fasted amplitude ratio		1.73 (1.49–1.86)	1.81 (1.31–2.35)	0.98

*Note:* ↑↓ Direction of severity for each questionnaire.

Abbreviations: μV, microvolts; cpm, cycles per minute.

Gastroparesis and FD subjects were, therefore, clinically indistinguishable across symptom severity, functional disability, psychometric profiles, and BSGM spectral metrics.

### 3.2 BSGM: Phenotype Subgroup Analysis

The 25 patients were defined and categorized a priori into three distinct phenotypes according to their spectral metrics and symptom profiles, regardless of FD or gastroparesis status, as follows:

- *BSGM Normal* (*n* = 10): indistinguishable spectral metrics from healthy controls
- *BSGM Delayed* (*n* = 8): delay in the onset of gastric activity postprandially defined by meal response ratio < 1 (ratio of amplitude in the first 2 h postprandially to the last 2 h).
- *BSGM Low Stability/Low Amplitude* (*n* = 7): have a GA‐RI < 0.25 and/or BMI‐Adjusted amplitude < 22 μv

There were no differences in age, BMI, or test quality measures between phenotypes (all *p*‐values > 0.05). BSGM metrics, symptoms, and questionnaire scores are provided in Table 5. The BSGM phenotype groups demonstrated significant differences in psychology and physical health metrics, including anxiety scores, which were worse for the *Low Stability/Low Amplitude* phenotype (59.5 [51.8–61.8]) than for *BSGM Delayed* (57.4 [47.5–59.5]) and *BSGM Normal* (43.5 [36.8–47.2], *p* = 0.03) phenotypes. A similar pattern emerged with Functional Disability scores, with the *Low Stability/Low Amplitude* phenotype reporting a higher impact on functional ability (21.5 [8.4–26.5]) than *BSGM Delayed* (18.5 [2.3–36.8]) and *BSGM Normal* (12.5 [3.0–24.0], *p* = 0.04) phenotypes. This pattern was also observed in the abdominal pain severity index scores (20.0 [11.3–27.3] vs. 21.0 [6.25–29.5] vs. 28.2 [3.75–33.0], *p* = 0.06), and quality of life (63.5 [45.3–85.7] vs. 59.5 [50.7–75.5] vs. 57.5 [46.3–76.3], *p* = 0.06), although not reaching statistical significance.

**TABLE 5:** Spectral data, total symptom burden, individual symptom scores, clinical symptoms, quality of life, functional disability, and mental well‐being questionnaire outcomes by phyotypes, BSGM normal, BSGM delayed, and low stability/low amplitude.

Variables (median [IQR])	Phenotypes			BSGM normal versus delayed versus LS/LA	BSGM normal versus BSGM delayed	BSGM normal versus LS/LA	BSGM delayed versus LS/LA
	BSGM normal	BSGM delayed	LS/LA				
	*N* = 10	*N* = 8	*N* = 7	*p*	*p*	*p*	*p*
BSGM metrics (median [IQR])							
PGF (cpm)	3	2.96	2.84	0.25	0.89	0.23	0.21
	(2.86–3.05)	(2.89–3.17)	(2.62–3.05)				
BMI‐adj amplitude (μV)	34.6	39.05	19.8	0.01	0.88	0.06	0.006
	(30.0–45.7)	(26.7–49.4)	(17.1–29.5)				
GA‐RI	0.45	0.45	0.19	0.003	0.63	< 0.001	< 0.001
	(0.31–0.52)	(0.33–0.57)	(0.16–0.19)				
ff‐AR	1.65	1.84	1.56	0.29	0.94	0.12	0.16
	(1.19–3.36)	(1.21–2.12)	(1.21–2.12)				
Meal response ratio	1.37	0.84	1.06	0.004	0.01	0.48	0.048
	(1.11–1.44)	(0.81–0.95)	(1.01–1.34)				
Symptoms (median [IQR])							
Total symptom burden score	18.35	25.1	33.2	0.08	0.76	0.055	0.15
	(5.7–27.9)	(10.5–27.5)	(17.1–38.7)				
Early satiety	4.5	3	6	0.72	0.52	0.35	0.71
	(2.5–6.0)	(0.25–5.7)	(0.0–8.0)				
Excessively full	0.9	2.55	3.3	0.49	0.48	0.09	0.57
	(0.07–3.2)	(0.25–5.7)	(1.3–7.5)				
Bloating	1.9	1.45	4	0.43	0.6	0.04	0.09
	(0.37–2.8)	(0.0–3.4)	(0.4–7.1)				
Heartburn	0.3	0.4	0.3	0.6	0.65	0.64	0.9
	(0.0–2.7)	(0.02–2.7)	(0.0–4.1)				
Nausea	1.4	3.5	5.5	0.04	0.38	0.04	0.26
	(0.07–4.1)	(0.15–6.9)	(4.8–9.1)				
Upper gut pain	1.35	2.05	4.9	0.06	0.11	0.03	0.16
	(0.0–5.2)	(0.0–6.2)	(1.6–5.7)				
Stomach burn	0	0.75	0	0.69	0.3	0.92	0.38
	(0.0–3.7)	(0.0–5.3)	(0.0–2.5)				
Symptoms, quality of life, functional disability, and psychometric questionnaires (median [IQR])							
Gastrointestinal symptoms	66.22	46.28	54.22	0.33	0.55	0.88	0.71
	(40.88–73.82)	(28.72–47.30)	(38.51–66.22)				
Nausea severity scale	15	25.5	29.5	0.57	0.44	0.32	0.81
	(2.2–25.7)	(14.3–30.5)	(20.0–32.0)				
Abdominal pain index	20	21	28.2	0.06	0.75	0.06	0.5
	(11.3–27.3)	(6.25–29.5)	(3.75–33.0)				
Functional disability index	12.5	18.5	21.5	0.04	0.087	0.042	0.73
	(3.0–24.0)	(2.3–36.8)	(8.7–26.5)				
Quality of life	63.5	59.5	57.5	0.06	0.09	0.06	0.72
	(45.3–85.7)	(50.7–75.5)	(46.3–76.3)				
Anxiety	43.5	57.4	59.5	0.03	0.09	0.03	0.5
	(36.8–47.2)	(47.5–59.5)	(51.8–61.8)				
Depression	43.4	52.3	50.7	0.56	0.1	0.33	0.88
	(37.7–56.7)	(40.7–63.3)	(43.9–65.6)				

Abbreviations: μV, microvolts; cpm, cycles per minute; ff‐AR, fed‐to‐fasted amplitude ratio; GA‐RI, gastric alimetry rhythm index; LS/LA, low stability/low amplitude; PGF, principal gastric frequency.

**FIGURE 3:**
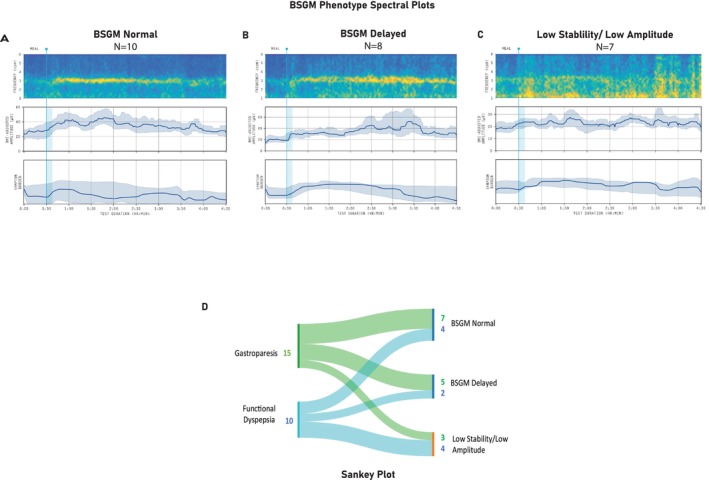
Average spectral and symptom data for each BSGM phenotype: BSGM Normal (A), Delayed (B), and Low Stability/Low Amplitude (C). The Sankey Plot (D) shows there was no relationship between clinical diagnoses (FD and gastroparesis) and the BSGM phenotypes (3D).

Within‐test symptom comparisons between the phenotype groups found that nausea was highest in the *Low Stability/Low Amplitude* group (5.5 vs. *BSGM Delayed* 3.5 vs. *BSGM Normal* 1.4, *p* = 0.04). The total symptom burden score was also higher in the *Low Stability/Low Amplitude* group (33.2 [17.1–38.7] vs. *BSGM Delayed* 25.1 [10.5–27.5] and *BSGM Normal* 18.3 [5–7‐27.9], although not reaching statistical significance [*p* = 0.08]). Pairwise analyses showed higher symptom severity in the *Low Stability/Low Amplitude* group compared to *BSGM Normal* for nausea (5.5 [4.8–9.1] vs. 1.4 [0.07–4.1], *p* = 0.04), upper gut pain (4.9 [1.6–5.7] vs. 1.35 [0.0–3.7], *p* = 0.03), and bloating (4.0 [0.4–7.1] vs. 1.9 [0.37–2.8], *p* = 0.04). The remaining symptoms did not differ between groups; however, there are visible differences in the symptom curves for each symptom by phenotype (Figure 3A–C).

Figure 3A–C displays the average spectral and symptom data for each BSGM phenotype. *BSGM Normal* (A) showed a high symptom burden that was present pre‐prandially and continued postprandially, being moderately meal‐responsive and with no correlation between the gastric amplitude and symptom curves (Spearman's correlation *r* = 0.11 *p* = 0.7 [95% CI −0.53 to 0.7]). *BSGM Delayed* (B) showed an increase in symptoms postprandially, which decreased as gastric amplitude increased (Spearman's correlation *r* = −0.26, *p* = 0.06, 95% CI −0.18 to 0.54). The *Low Stability/Low Amplitude* phenotype (C) showed a relatively high symptom burden pre‐prandially, which remained continuous throughout the test (total symptom burden score 33.2) and with symptom curves uncorrelated with gastric amplitude (Spearman's correlation *r* = 0.21, *p =* 0.65 [95% CI −0.78 to 0.55]).

There was no relationship between the clinical diagnoses of FD and gastroparesis and the physiological phenotypes revealed by BSGM, as demonstrated by the Sankey Plot in Figure 3D.

## 4 Discussion

This study applied a new BSGM technique (Gastric Alimetry) in an adolescent population to determine whether meaningful new pediatric disease groupings could be delineated in FD and gastroparesis. The results demonstrated that FD and gastroparesis patients could not be separated by their symptoms, quality of life, functional disability scores, health psychology metrics, or BSGM metrics. In contrast, three distinct BSGM phenotypes emerged from the spectral analysis, showing meaningful clinical differences across most of these domains. The clinical diagnosis of FD or gastroparesis showed no relationship to the three BSGM phenotypes, indicating that they potentially constitute novel disease categories.

Our findings, supported by other research, found substantial overlap between FD and gastroparesis regarding symptom patterns, quality of life, and mental well‐being [10, 36]. The overlap between FD and gastroparesis poses difficulties for personalized care, particularly for predicting patient responses to management options. Consequently, there is an element of trial and error in current clinical decisions and patient care pathways [37, 38], which appears unavoidable without improved diagnostic tools to provide improved clinical biomarkers. BSGM introduces objective phenotyping and symptom profiling and thus offers an alternate approach or a secondary diagnostic layer to GET [9, 18, 39].

A known weakness of GET is that it does not dependably capture neuromuscular pathologies, such as injury to the interstitial cell of Cajal (ICC) networks, which can lead to dysrhythmic gastric myoelectrical activity [40]. A primary motivation for developing BSGM was to resolve this gap by introducing a test more specific for neuromuscular disorders, considered to be characterized here by the Low GA‐RI/Low Amplitude phenotype [18, 41]. In this first BSGM study in adolescents, this phenotype was identified in both gastroparesis and FD subjects and was notable for its more severe symptoms (particularly nausea), poorer health psychology, physical health, and lower quality of life. These factors indicate that this phenotype likely distinguishes a more severely affected subset of pediatric patients requiring more intensive management approaches.

Patients with high symptom burden, unrelated to gastric amplitude, plausibly indicate a gut‐brain axis relationship (*BSGM Normal* phenotype) [42]. In adults, research has found that patients in this category often experience higher psychological comorbidities [37, 43, 44, 45]; however, in our adolescent study, anxiety and depression scores in this group were similar to the population average, indicating that other factors should also be considered [46, 47]. Patients with the *BSGM Delayed* group constitute a new category, with a temporal disconnect between the meal challenge and subsequent meal response (amplitude increase), with symptoms occurring predominantly during the lag phase (no gastric activity). The delayed response phenotype of BSGM can be linked to altered gastric postprandial physiology, either because of altered CNS response to a meal, altered autonomic function, altered gastric accommodation, or delayed hormonal response to a meal. Further research could provide valuable insights to enhance pathophysiological understanding and diagnosis and improve treatment planning. The finding that seven patients diagnosed with gastroparesis had a normal spectral outcome (*BSGM Normal* phenotype) is significant and could indicate a focus on alternative pathophysiology, including pyloric resistance [48].

This study also included the recruitment of the first cohort of adolescent healthy control subjects. This data was applied to compare adolescent and adult control data collected from a separate study [32], demonstrating that adult reference intervals can provide an acceptable provisional BSGM interpretation framework. While this qualifying step enabled the delineation of the first pediatric spectral groupings, we recommend that age‐specific pediatric reference intervals be prioritized in the future. This recommendation is supported by the observation of a modestly reduced average GA‐RI metric and a noticeably shorter meal response duration in adolescent controls from this initial study. These findings may be influenced by a higher metabolic rate in adolescents, leading to a quicker return to a fasting state [49, 50], which was illustrated by the lower GA‐RI occurring late in the test.

Several limitations are also acknowledged. While our sample size was sufficient for delineating novel pediatric disorder subgroups, further studies with larger patient groups are now desirable to confirm and expand the outcomes reported here. This study focused on FD confirmed by Rome IV and a normal gastric emptying scintigraphy result and gastroparesis patients only. Given the prevalence of common pediatric DGBI conditions such as functional abdominal pain, chronic nausea, and vomiting [1], expanding BSGM research into more conditions is important. In addition, studies linking the newly defined subgroups to therapeutic outcomes comprise a critical next step to further defining their clinical importance and utility. The limited male representation in the patient group is unsurprising, as the prevalence of these disorders is higher in females [51, 52, 53]. Nevertheless, increasing male representation in these studies is important.

## 5 Conclusion

This first study of BSGM in a pediatric population has identified novel subgroups in adolescents with chronic gastroduodenal symptoms. These groups showed meaningful clinical differences, which were absent when comparing the current FD and gastroparesis diagnoses. These findings indicate that BSGM can provide valuable data to classify disease phenotypes within these complex conditions, which could support differential treatment approaches, follow‐ups, and monitoring.

## Author Contributions

G.H. designed the research study, collected and analyzed the data, and wrote the manuscript. G.O., H.M., A.G. and C.K. contributed to the study design, advice and manuscript revisions. X.B. collected data and contributed to manuscript revisions. G.S. provided advice on the study and analysis and contributed to manuscript revisions. C.N.A., A.B., and S.C. provided study advice and contributed to manuscript revisions. All authors peer‐reviewed and approved the final version of the manuscript.

## Conflicts of Interest

G.O.G. and A.G. hold grants and intellectual property in gastrointestinal electrophysiology. G.S., S.C., G.O.G., A.G., and C.N.A. are Alimetry's shareholders and employees. G.O.G. is a Director at The Insides Company. The remaining authors have no conflicts of interest to declare.

## Supporting information


Figure S1.

Figure S2.

## Data Availability

Data sharing and data use are governed by the Aotearoa|New Zealand Health and Disability Ethics Committee. The majority of the data is available in the manuscript; however, all requests for additional data can be made to the corresponding author. Requests will be granted if the proposed use aligns with the ethical approval for the study and relevant ethical approvals have been obtained, including approval from a New Zealand Ethical Committee and Children's Hospital of Philadelphia IRB.
