# Supplementary figures and images for: Body Surface Gastric Mapping Delineates Specific Patient Phenotypes in Adolescents With Functional Dyspepsia and Gastroparesis
Source: Neurogastroenterol Motil. 2025 Mar 19;37(6):e70018. doi: 10.1111/nmo.70018 (PMC12075902; doi:10.1111/nmo.70018)

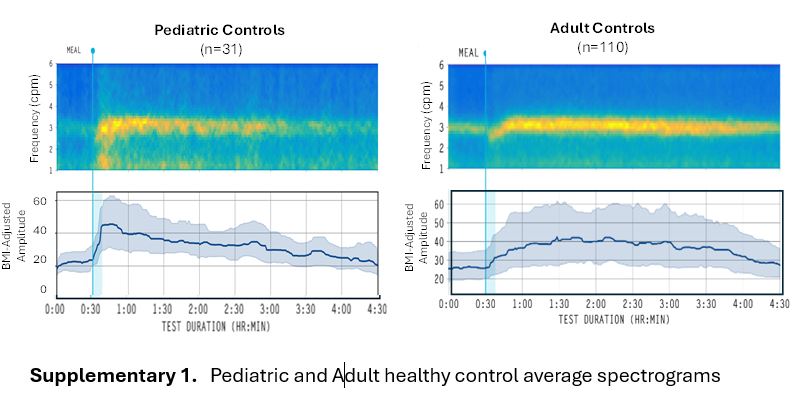

Supplement: Supplementary file 1 — Figure S1. [file NMO-37-e70018-s002.jpg]

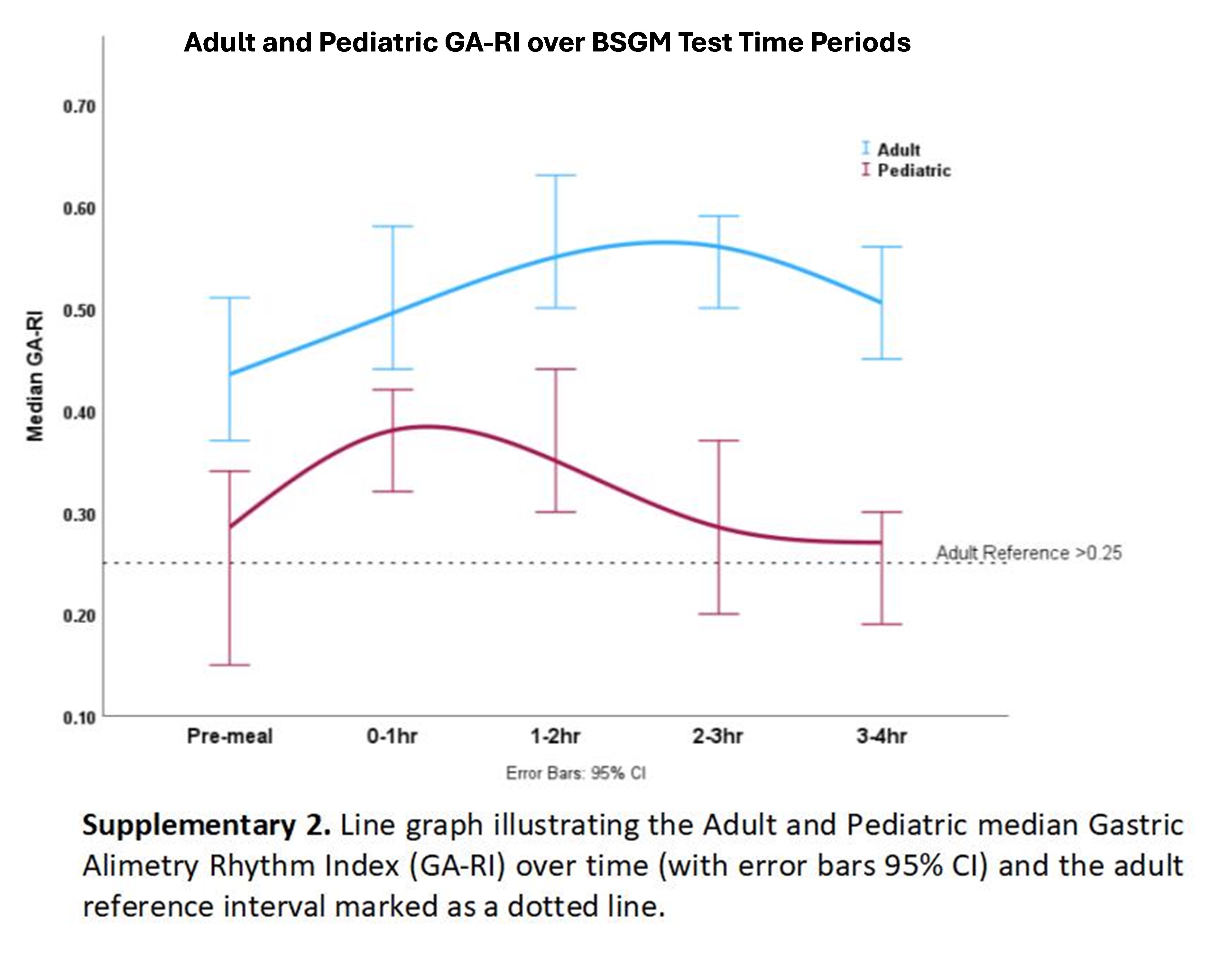

Supplement: Supplementary file 2 — Figure S2. [file NMO-37-e70018-s001.jpg]
